# Re-Auditing of Quality Improvement Project in Psychiatric Liaison Service for Evaluation of the Ageless Service Model

**DOI:** 10.1192/bjo.2026.11597

**Published:** 2026-06-30

**Authors:** Shazia Kashif, Shalina Ramsewak, Shruti Lodhi

**Affiliations:** Surrey and Borders Partnership NHS Trust, Camberley, United Kingdom

## Abstract

**Aims:**

This re-audit aimed to evaluate the Ageless Psychiatric Liaison Service (PLS) by: (1) assessing older adult (OA) and working age adult (WAA) referrals in terms of source, triage, assessments, and outcomes; (2) evaluating psychiatric admissions, including reasons for admission, senior medic involvement, observation levels, medical clearance, and bed management; and (3) appraising alternatives to admission, such as referrals to Crisis House and Home Treatment Teams (HTT).

**Methods:**

A retrospective review was conducted over three months (April–June 2025) using electronic psychiatric (SystmOne) and hospital (EPIC) records. A random sample of 50 patients (25 OA, 25 WAA) was selected, with two young-onset dementia cases included in the OA group. Data were extracted systematically using a structured Excel sheet capturing referral details, clinical assessments, management, and outcomes.

**Results:**

WAA presentations predominantly involved acute psychiatric crises, including self-harm and suicidal ideation, with short hospital stays (mostly 1–2 days) and low observation requirements. Referrals were primarily crisis-driven, with 88% medically optimized for discharge at the time of referral, and were largely managed through a nurse-led model by Band 6 and 7 nurses. Most WAA patients were discharged to community services, including HTT, CMHRS or GP with only a small proportion requiring acute hospital admission.

In contrast, OAA presentations were more complex, involving cognitive, behavioural, and medical comorbidities, resulting in longer and more variable hospital stays and needing one-to-one nursing in nearly half of cases. Management was multidisciplinary, involving old age specialists, Band 7 nurses, and PLS medics in 92% of cases. Half of OAA patients were referred to CMHT-OP for ongoing management, while 20% awaited psychiatric admission due to limited bed availability. Delays in transfer to psychiatric beds ranged from 23 hours to 21 days, reflecting a scarcity of psychiatric beds.

**Conclusion:**

The re-audit highlighted effective nurse-led crisis management for WAA, whereas OAA patients presented with complex needs requiring multidisciplinary input and longer hospital stays. The lack of an established older adult crisis pathway contributes to reliance on CMHT-OP and prolonged admission. These findings underscore the need for tailored crisis pathways and resource allocation to better support the complex needs of older adults while confirming the efficiency of current approaches for working-age patients. The PLS ageless model and mix of clinicians of various experience and background need to reflect the patient population that is served. There is a clear need for WAA and OA specialism within the ageless model.

